# Markers of Sleep-Disordered Breathing and Metabolic Syndrome in a Multiethnic Sample of US Adults: Results from the National Health and Nutrition Examination Survey 2005–2008

**DOI:** 10.1155/2012/630802

**Published:** 2012-04-22

**Authors:** Charumathi Sabanayagam, Ruoxin Zhang, Anoop Shankar

**Affiliations:** ^1^Department of Community Medicine, West Virginia University School of Medicine, Robert C. Byrd Health Sciences Center, 1 Medical Center Drive, P.O. Box 9190, Morgantown, WV 26505-9190, USA; ^2^Singapore Eye Research Institute, Singapore National Eye Centre, 11 Third Hospital Avenue No. 05-00, Singapore 168751

## Abstract

Previous studies have documented an association between markers of sleep-disordered breathing (SDB) and metabolic syndrome. However, it is not clear if there are gender or ethnic differences in this association. We examined 6,122 participants aged ≥20 years from the National Health and Nutrition Examination Survey 2005–08. Metabolic syndrome was defined as the presence of ≥3 of the following components: (1) abdominal obesity, (2) elevated blood triglycerides, (3) low HDL cholesterol, (4) high BP, and (5) hyperglycemia. SDB severity was defined based on an additive summary score including sleep duration, snoring, snorting, and daytime sleepiness. We found that short sleep duration, snoring, snorting, daytime sleepiness and the summary SDB score were significantly associated with metabolic syndrome independent of potential confounders. Compared to those without any sleep disturbance, the multivariable odds ratio (OR) (95% confidence interval [CI]) of metabolic syndrome among those with three or more sleep disturbances was 3.92 (2.98–5.16). In subgroup analyses, this association was consistently present among men and women and all race-ethnic groups. In summary, SDB was independently associated with metabolic syndrome in a nationally representative sample of US adults.

## 1. Introduction

Sleep-disordered breathing (SDB) is a common condition affecting 1 in 5 adults in the USA and is associated with metabolic abnormalities including diabetes [1, 2], hypertension [3], and obesity [4]. Metabolic syndrome, a constellation of metabolic abnormalities including obesity, high blood pressure, dyslipidemia, and hyperglycemia is an established risk factor for diabetes and cardiovascular disease [5]. Previous studies have documented an association between a variety of markers of SDB including snoring [6, 7], daytime sleepiness [7], or sleep duration [8] and metabolic syndrome or its components in the general population [9]. However, most of the studies that examined the association between SDB and metabolic syndrome assessed either a single marker of SDB [6, 8, 9] or separate components of metabolic syndrome [9].

Studies have also documented gender [10, 11] or ethnic differences [12, 13] in the prevalence of SDB. Men [10, 11] and African Americans [12, 13] were reported to have increased prevalence of SDB in the US. Similar differences in the prevalence of metabolic syndrome have been documented. In the National Health and Nutrition Examination Survey (NHANES) 2003–2006, non-Hispanic black men and women were reported to have higher prevalence of metabolic syndrome than non-Hispanic white men and women [14]. Further, the prevalence of components of metabolic syndrome including diabetes, hypertension and obesity is also reported to be higher among African Americans [15]. Few previous studies have reported an association between SDB and metabolic syndrome across gender or ethnic groups using objectively defined measures of SDB. In the current study, we aimed to examine the association between subjective measures of SDB defined by a combination of snoring, snorting, daytime sleepiness and sleep duration, and metabolic syndrome in a nationally representative sample of US adults. In addition, we also examined the association between subjective markers of SDB and metabolic syndrome stratified by gender and ethnic groups.

## 2. Methods

The data for this study is derived from the National Health and Nutrition Examination Survey 2005-06 and 2007-08. Detailed description of NHANES study design and methods are available elsewhere [16, 17]. In brief, the NHANES survey included a stratified multistage probability sample representative of the civilian noninstitutionalized US population. Selection was based on counties, blocks, households, and individuals within households and included oversampling of non-Hispanic blacks and Mexican Americans in order to provide stable estimates of these groups. We restricted our study sample to participants aged greater than 20 years. Questions on sleep were first included in the NHANES in 2005-06. Of the 10,914 participants with information on sleep variables, after excluding those with pregnancy (*n* = 393), prevalent cardiovascular disease (*n* = 1, 275), and those with missing information on plasma glucose, sleep variables (*n* = 2, 109), or variables included in the multivariable model (*n* = 1015), 6,122 were available for the final analysis.

### 2.1. Assessment and Definition of Metabolic Syndrome Components

 Metabolic syndrome was defined based on AHA-NHLBI [18] revised definition of ATP-III guidelines as the presence of 3 or more of the following components: (1) abdominal obesity, waist circumference >102 cm in men and 88 cm in women, (2) elevated blood triglycerides, ≥150 mg/dL, (3) low high-density lipoprotein (HDL) cholesterol, <40 mg/dL in men and <50 mg/dL in women, (4) high blood pressure (BP), ≥130/85 mm Hg or use of BP medications, and (5) hyperglycemia defined as a fasting serum glucose ≥100 mg/dL or on drug treatment for elevated glucose. 

Waist circumference was measured to the nearest 0.1 cm at minimal respiration at the end of normal expiration with a steel measuring tape placed at the high point of the iliac crest when the participant was in a standing position. BP was measured using a mercury sphygmomanometer, and an average of three measurements was taken as the systolic and diastolic BP value. 

Fasting plasma glucose levels were measured using hexokinase enzymatic method on Roche/Hitachi Modular P Chemistry Analyzer at the Fairview Medical Center Laboratory at the University of Minnesota, Minneapolis Minnesota. Serum total cholesterol, HDL cholesterol, and triglycerides were measured enzymatically using the Roche Hitachi 717 in 2005, Roche Hitachi 717 and 912 in 2006, and Roche Modular P chemistry analyzer in 2007-2008. Detailed description about the blood collection, processing and quality control checks are provided in the Laboratory Procedures Manual [19, 20].

### 2.2. Assessment of Exposure

 A questionnaire on sleep habits based on validated questions from previous epidemiological studies was introduced in NHANES from 2005 till 2008 [21]. SDB was assessed from a set of questions on sleep habits including “How much sleep do you usually get at night on weekdays or workdays?,” “In the past 12 months, how often did you snore while you were sleeping?,” “In the past 12 months, how often did you snort, gasp or stop breathing while you were asleep?,” and “In the past month, how often did you feel excessively or overly sleepy during the day?” Based on the responses to the above questions, we created four sleep variables: sleep duration, snoring, snorting, and daytime sleepiness. Sleep duration coded in hours was categorized into ≤5, 6, 7, 8 and ≥9 h. Snoring and snorting variables were categorized into 0–2 nights/week, 3-4 nights/week, and 5 or more nights/week. Daytime sleepiness was categorized into 0-1 time/month, 2–4 times/month, and 5 or more times/month. 

Since each of these sleep items were significantly correlated (with Pearson correlation coefficients ranging from 0.2 to 0.6; *P* < 0.0001), we also created an SDB summary score that indicates both the number of sleep items and the severity. For the summary score, we first dichotomized the individual variables based on their clinical significance and previous literature. [12, 22, 23], A score of 1 was assigned separately if the participants report sleep duration of ≤5, snoring at least 3-4 nights/week, snorting at least 3-4 nights/week, and daytime sleepiness at least 5 times/month. The summary score ranged from 0 to 4 corresponding to no sleep disturbance to coexistence of all 4 sleep disturbances. Diagnosed sleep apnea was assessed from two questions, “Have you ever been told by a doctor or other health professional that you have a sleep disorder?” and among those who answered affirmatively, “What was the sleep disorder?” with the response categorized into sleep apnea, insomnia, restless legs, and others. Participants who reported sleep apnea were classified to have diagnosed sleep apnea.

### 2.3. Assessment of Covariates

Information on age, gender, race/ethnicity, smoking status, alcohol intake (g/day), level of education, history of oral hypoglycemic intake or insulin administration, and antihypertensive medication use was obtained during a standardized questionnaire at home interview. Educational attainment was categorized into less than high school graduate, high school graduate, and more than high school graduate. Individuals who had smoked <100 cigarettes during their lifetime were considered never smokers, those who had smoked ≥100 cigarettes lifetime and currently not smoking were considered former smokers, and those who had smoked ≥100 cigarettes lifetime and currently smoking were considered current smokers. Alcohol consumption was categorized into never, former, current moderate, and current heavy drinker based on questionnaire response. Moderate physical activity was defined as engaging in moderate-intensity sports, fitness, or recreational activities that cause a small increase in breathing or heart rate such as brisk walking, bicycling, swimming, or golf for at least 10 minutes continuously in a typical week. Depression was assessed using the Patient Health Questionnaire (PHQ-9), a well-validated 9-item screening tool that asks questions about the frequency of symptoms of depression over the past 2 weeks [24]. Depression was defined as a PHQ-9 score of 10 or higher, a validated cut-point commonly used in clinical studies [24].

### 2.4. Statistical Analysis

We used analysis of covariance to estimate mean levels of each component of metabolic syndrome across categories of summary sleep score. We examined the association between categories of individual sleep variables, including snoring, snorting, daytime sleepiness and sleep duration, and metabolic syndrome in two multivariable models. In the first model, we adjusted for age (years) and sex (female, male). In the second model, we additionally adjusted for race-ethnicity (non-Hispanic whites, non-Hispanic blacks, Hispanic-Americans, others), education (<high school, high school, >high school), smoking (never, former, current), current alcohol consumption (never, former, current moderate and current heavy drinker), and moderate physical activity (absent, present). 

To examine the overall effect of SDB markers on metabolic syndrome, we created an additive summary SDB score and examined the association between this summary SDB score and metabolic syndrome in separate analyses. Tests for trend were performed using the categories of individual sleep variables and the summary score as an ordinal variable in the corresponding multivariable models. To examine the consistency of the association, we performed subgroup analyses stratified by sex and race-ethnicity. Interactions between SDB summary score and gender or race/ethnicity were assessed by introducing cross-product interaction terms in the corresponding multivariable models. All analyses were weighted to account for the unequal probabilities of selection, oversampling, and nonresponse using SUDAAN (version 8.0; Research Triangle Institute, Research Triangle Park, NC) and SAS (version 9.1.; SAS Institute, Cary, NC) software. In a supplementary analysis, to assess the validity of our self-reported sleep items and SDB summary score, we examined the association between SDB summary score and diagnosed sleep apnea in the multivariable model. In a second analysis, we examined the association between diagnosed sleep apnea and metabolic syndrome. 

## 3. Results

 The weighted prevalence of metabolic syndrome in the study population was 37.3%.  Table 1 shows the characteristics of the study population. The mean age of the study population was 44.6 years and 50% of them were women. Majority of the participants were non-Hispanic whites (73.1%) and current drinkers (75.1%). Fifty-nine percent were high school or above educated and 23.6% were current smokers. 

Table 2 shows the association between the various sleep variables and metabolic syndrome. Compared to sleep duration of 7 h (referent), sleep duration ≤5 h was positively associated with metabolic syndrome in both the age, sex-adjusted, and the multivariable models. Similarly, snoring at least 3-4 nights/week, snorting at least 3-4 nights/week, and daytime sleepiness at least 2–4 times/month were all significantly associated with metabolic syndrome in both the age, sex-adjusted, and the multivariable models. Finally, to examine the effect of cooccurrence of sleep disturbances on metabolic syndrome, we used the SDB summary score. A significant graded association was observed between increasing SDB summary score and metabolic syndrome (*P*-trend <0.0001). 

Table 3 shows the association between the summary SDB score and metabolic syndrome stratified by gender. Similar to the main results in Table 2, a positive association was observed between the summary SDB score and metabolic syndrome in both men and women; however, the association was stronger in women (*P*-interaction = 0.002).  Table 4 shows the association between the summary SDB score and metabolic syndrome stratified by race-ethnicity. Consistent with the main results in Table 2, the association between summary SDB score and metabolic syndrome was found to be consistently present in all race-ethnic groups (*P*-interaction = 0.6). 

 In a supplementary analysis, examining the association between SDB summary score and diagnosed sleep apnea, compared to those with SDB summary score of 0, the multivariable odds ratio (OR) (95% confidence interval [CI]) of sleep apnea among those with SDB summary score ≥3 was 9.52 (5.73–15.80). In a second analysis, we examined the association between diagnosed sleep apnea and metabolic syndrome. 4.3% of the participants reported having diagnosed sleep apnea. Similar to the individual sleep variables, a significant positive association was observed between diagnosed sleep apnea and metabolic syndrome. Compared to those without diagnosed sleep apnea, the multivariable odds ratio (OR) (95% confidence interval [CI]) of metabolic syndrome among those with diagnosed sleep apnea was 3.07 (1.88–5.00).

## 4. Discussion

 In a representative sample of US adults, we found that markers of SDB including short sleep duration (≤5 h), snoring at least 3-4 nights/week, snorting at least 3-4 nights/week, and daytime sleepiness at least 2–4 times/month were associated with metabolic syndrome independent of age, sex, race-ethnicity, education, smoking, alcohol consumption, and physical activity. Further, an *ad hoc* summary SDB score that provided a measure of the severity of SDB by counting the cooccurrence of these four sleep disturbances showed that compared to those without any SDB markers, those with three or more SDB markers had nearly fourfold odds of having metabolic syndrome. In subgroup analyses, the SDB-metabolic syndrome association was found to be similar by sex or race-ethnicity. 

 In the current study, we found that short sleep duration, snoring, and snorting were associated with metabolic syndrome consistent with previous studies [7, 8]. Troxel et al. has shown that snoring was associated with 3-year incidence of metabolic syndrome in a community-based sample of US adults [7]. Leineweber et al. have shown a positive association between snoring and metabolic syndrome in middle-aged Swedish women [6]. Hall et al. reported a positive association between both short and long duration of sleep and metabolic syndrome in a community-based sample of middle-aged adults [8].

Few studies have examined the relation between SDB and metabolic syndrome by gender or race/ethnicity using objective measures of SDB. Sasanabe et al. in a hospital-based case-control study in Japan observed a positive association between SDB and metabolic syndrome in both men and women; however, the association in women was short of statistical significance [25]. Nock et al. in a clinic-based study in the US observed a positive association between SDB and metabolic syndrome that was consistent across gender and race-ethnic groups [26]. Nieto et al. reported a positive association between SDB and metabolic syndrome in a community-based sample comprising almost exclusively white participants (96% whites) in the USA [27].

 It is plausible that SDB may play a role in metabolic syndrome through several mechanisms. Intermittent hypoxia and sleep fragmentation in SDB leads to insulin resistance and metabolic alterations through activation of hypothalamo-pituitary-adrenal axis [28], excessive accumulation of reactive oxygen species [29], enhanced production of proinflammatory cytokines [30], and upregulation of leptin secretion [31]. Studies have also shown that SDB is associated with obesity, diabetes, hypertension, and lipid abnormalities [32].

 The large multiethnic sample with rich information on potential confounders and the rigorous methodology of data collection in NHANES are the main strengths of the study. Our study has some limitations. First, assessment of SDB utilizing self-reported sleep items with no psychometric support for their questionnaire items or any data to indicate that grouping the items together was psychometrically sound might have introduced a bias. Second, we cannot exclude the possibility of residual confounding due to measurement error resulting from our less than comprehensive assessment of physical activity. Third, our cross-sectional study design limits making temporal associations between SDB and metabolic syndrome. 

In conclusion, in a nationally representative sample of US adults, we found that markers of SDB, including short sleep duration, snoring, snorting, and daytime sleepiness were associated with metabolic syndrome independent of potential confounders. This association was consistently present in men and women and across ethnic groups. If confirmed by future prospective studies, our findings may have important clinical implications for screening and managing sleep disorders in adults with metabolic syndrome.

## Figures and Tables

**Table 1:** Baseline characteristics of the study population.

Characteristics	Mean values ± standard error (SE) or number (percentages) (*n* = 6122)
Women (%)	2950 (50.1)
Age (years)	44.63 ± 0.46
Race/Ethnicity (%)	
Non-Hispanic Whites	3047 (73.1)
Non-Hispanic Blacks	1214 (9.7)
Mexican Americans	1139 (7.9)
Others	722 (9.3)
Education categories (%)	
Below high school	1622 (16.7)
High school	1481 (24.3)
Above high school	3019 (59.0)
Smoking (%)	
Never smoker	3242 (52.6)
Former smoker	1461 (23.8)
Current smoker	1419 (23.6)
Alcohol intake, (%)	
Never drinker	780 (10.1)
Former drinker	1104 (14.9)
Current moderate drinker	2529 (46.4)
Current heavy drinker	1709 (28.6)
Moderate physical activity (%)	2870 (53.9)
Total cholesterol (mg/dL)	0.38 ± 0.01
Waist circumference (cm)	96.95 ± 0.42
Triglycerides (mg/dL)	157.98 ± 2.5
HDL-cholesterol (mg/dL)	53.16 ± 0.34
Systolic blood pressure (mm Hg)	120.48 ± 0.34
Diastolic blood pressure (mm Hg)	70.92 ± 0.29
Fasting glucose (mg/dL)	104.22 ± 0.61

**Table 2:** Association between sleep disordered breathing variables and metabolic syndrome.

Sleep variables	Sample size (Metabolic syndrome %)	Age, sex-adjusted odds ratio (95% CI)	Multivariable adjusted* odds ratio (95% CI)
Sleep duration (hours)			
≤5 hrs	935 (40.3)	1.31 (1.04–1.64)	1.24 (0.98–1.57)
6 hrs	1412 (38.8)	1.12 (0.94–1.34)	1.11 (0.93–1.34)
7 hrs	1777 (36.6)	1 (referent)	1 (referent)
8 hrs	1621 (35.7)	0.94 (0.78–1.14)	0.92 (0.76–1.11)
≥9 hrs	377 (35.9)	1.01 (0.73–1.40)	0.97 (0.69–1.36)
*P*-trend		0.01	0.02
Snoring (nights/week)			
0–2	2952 (27.2)	1 (referent)	1 (referent)
3-4	1188 (39.2)	1.67 (1.42–1.98)	1.69 (1.42–2.00)
≥5	1982 (51.7)	2.78 (2.38–3.25)	2.77 (2.38–3.24)
*P*-trend		<0.0001	<0.0001
Snorting (nights/week)			
0–2	5370 (35.0)	1 (referent)	1 (referent)
3-4	394 (47.7)	1.54 (1.16–2.06)	1.52 (1.16–1.98)
≥5	358 (60.2)	2.70 (2.21–3.30)	2.59 (2.13–3.14)
*P*-trend		<0.0001	<0.0001
Daytime sleepiness (times/month)			
0-1	3547 (36.1)	1 (referent)	1 (referent)
2–4	1583 (37.0)	1.15 (1.01–1.31)	1.15 (1.01–1.32)
≥5	992 (41.8)	1.54 (1.31–1.81)	1.44 (1.22–1.70)
*P*-trend		<0.0001	<0.0001
Sleep summary score			
0	2210 (26.9)	1 (referent)	1 (referent)
1	2424 (40.1)	1.80 (1.47–2.19)	1.80 (1.48–2.19)
2	1100 (46.7)	2.41 (2.03–2.87)	2.38 (1.99–2.85)
≥3	388 (56.8)	4.00 (3.05–5.24)	3.82 (2.89–5.04)
*P*-trend		<0.0001	<0.0001

*Adjusted for age (years), gender (male, female), race/ethnicity (non-Hispanic whites, non-Hispanic blacks, Mexican Americans, others), education (below high school, high school, above high school), smoking (never smoker, former smoker, current smoker), alcohol intake (never, former, current moderate, current heavy), moderate physical activity (absent, present), and depression (absent, present).

**Table 3:** Association between sleep variables and metabolic syndrome levels by gender.

Sleep summary score	Sample size (Metabolic syndrome %)	Age-adjusted OR (95% CI)	Multivariable adjusted* OR (95% CI)
Men			
0	1011 (28.5)	1 (referent)	1 (referent)
1	1298 (38.4)	1.54 (1.17–2.02)	1.57 (1.19–2.07)
2	645 (46.0)	2.09 (1.63–2.68)	2.18 (1.69–2.82)
≥3	218 (50.3)	2.74 (1.98–3.79)	2.91 (2.12–4.01)
*P*-trend		<0.0001	<0.0001
Women			
0	1199 (25.7)	1 (referent)	1 (referent)
1	1126 (41.9)	2.03 (1.56–2.64)	2.01 (1.55–2.59)
2	455 (47.8)	2.72 (2.19–3.38)	2.52 (2.04–3.11)
≥3	170 (65.0)	6.14 (4.16–9.07)	5.28 (3.54–7.87)
*P*-trend		<0.0001	<0.0001

*Adjusted for age (years), race/ethnicity (non-Hispanic whites, non-Hispanic blacks, Mexican Americans, others), education (below high school, high school, above high school), smoking (never smoker, former smoker, current smoker), alcohol intake (never, former, current moderate, current heavy), moderate physical activity (absent, present), and depression (absent, present).

**Table 4:** Association between sleep variables and metabolic syndrome levels by race/ethnicity.

Sleep summary score	Sample Size (Metabolic syndrome %)	Age, sex-adjusted odds ratio (95% CI)	Multivariable adjusted* odds ratio (95% CI)
Non-Hispanic Whites			
0	1143 (28.9)	1 (referent)	1 (referent)
1	1179 (41.9)	1.76 (1.36–2.29)	1.76 (1.36–2.28)
2	536 (48.0)	2.31 (1.82–2.93)	2.25 (1.76–2.87)
≥3	189 (58.6)	4.08 (2.94–5.67)	3.71 (2.69–5.14)
*P*-trend		<0.0001	<0.0001
Non-Hispanic Blacks			
0	413 (21.2)	1 (referent)	1 (referent)
1	462 (33.5)	1.87 (1.35–2.59)	1.85 (1.33–2.58)
2	239 (39.6)	2.62 (1.60–4.29)	2.60 (1.60–4.23)
≥3	100 (45.0)	3.43 (2.00–5.91)	3.32 (1.90–5.79)
*P*-trend		<0.0001	<0.0001
Mexican Americans and others			
0	654 (21.3)	1 (referent)	1 (referent)
1	783 (36.1)	1.86 (1.26–2.75)	1.81 (1.22–2.69)
2	325 (45.7)	2.88 (1.96–4.23)	2.78 (1.87–4.14)
≥3	99 (59.2)	4.38 (4.49–7.70)	4.36 (2.45–7.75)
*P*-trend		<0.0001	<0.0001

*Adjusted for age (years), gender (male, female), education (below high school, high school, above high school), smoking (never smoker, former smoker, current smoker), alcohol intake (never, former, current moderate, current heavy), moderate physical activity (absent, present), and depression (absent, present).
